# Spinal Flexibility Is an Important Factor for Improvement in Spinal and Knee Alignment after Total Knee Arthroplasty: Evaluation Using a Whole Body EOS System

**DOI:** 10.3390/jcm9113498

**Published:** 2020-10-29

**Authors:** Seong Chan Kim, Joo Sung Kim, Han Gyeol Choi, Tae Woo Kim, Yong Seuk Lee

**Affiliations:** 1 Department of Orthopaedic Surgery, Seoul National University College of Medicine, Seoul National University Bundang Hospital, Gyeonggi-do 13620, Korea; circlein@naver.com (S.C.K.); mdndkfc@gmail.com (J.S.K.); meinmed87@naver.com (H.G.C.); 2 Department of Orthopedic Surgery, Jeju Hyein Foundation Hankook General Hospital, Jeju-do 63183, Korea; 3 Department of Orthopedic Surgery, Seoul National University College of Medicine, Seoul National University Boramae Medical Center, Seoul 07061, Korea; orthopassion@naver.com

**Keywords:** knee, osteoarthritis, total knee arthroplasty, spinal flexibility, EOS, sagittal alignment

## Abstract

The purposes of this study were (1) to evaluate the relationship between lumbosacral flexibility and the effects of total knee arthroplasty (TKA) on whole-body alignment; and (2) to determine the prerequisites of the adjacent joints for successful TKA. A total of 116 patients (156 cases) who had whole-body X-ray and flexion-extension lumbar radiograph available were enrolled. For the sagittal alignment evaluation, hip–knee–ankle (HKA) angle, pelvic tilt (PT), sacral slope (SS), lumbar lordosis (LL), thoracic kyphosis (TK), and C7 plumb line-sacrum distance (SVA) were evaluated on the whole-body radiographs. Lumbar flexibility (LF) was evaluated using the flexion-extension lumbar radiographs, and pelvic flexibility (PF) was evaluated using the pelvic incidence (PI). The disparities in the knee joint between postoperative passive motion and weight-bearing posture were assessed. LF was significantly correlated with ΔLL and ΔSVA (LL: *p* = 0.039, SVA: *p* = 0.040; Pearson correlation coefficient (PCC): −0.206 and 0.205, respectively). There were correlations between PF and ΔSS (*p* < 0.001, PCC: −0.362), and between the disparity and LF (*p* = 0.005, PCC = −0.275). Linear regression analysis demonstrated that LF was significantly associated with the presence of disparity (*p* = 0.005, β = −0.205). LF is an important factor for improved spinal and lower limb alignment after TKA. Additionally, reduced LF may result in knee joint disparity between passive extension and standing extension status. Therefore, surgeons should consider spinopelvic alignment, including lower limb alignment preoperatively, to be able to predict possible changes in whole-body alignment following TKA.

## 1. Introduction

As the aging population increases, many patients experience multi-site degenerative changes, with the knee and spine being the two most commonly affected sites. Therefore, it is common to encounter patients who have combined knee and spinal problems, such as deformed knee osteoarthritis and spinal degenerative kyphosis [1,2,3]. In cases of multi-joint problems, the treatment strategy should be established by considering the effect between each joint. Total knee arthroplasty (TKA) is a well-established procedure in osteoarthritic knee patients [4,5,6] and TKA patients commonly present with spinal problems, such as lower back pain (LBP). [2] Although many patients have improved spinal problems after TKA, some complain of aggravated spinal problems or recurrence of knee flexion deformity for the standing body balance, even if it was previously solved with TKA. This may imply that multiple joints can affect each other; therefore, it is very important to consider these effects after TKA.

In general, knee surgeons have primarily focused on knee problems and have rarely considered the effects of other joints when performing TKA. However, as mentioned above, poor outcomes after TKA can often be predicted because spinal problems can be aggravated or a negative effect can be imparted on the knee joint, such as recurrence of flexion deformity. Therefore, it is important to assess the whole-body before TKA, and whether patients have any fixed or detrimental problems likely to affect the outcome of TKA. The EOS^TM^ whole-body X-ray system was introduced as a method of whole-body alignment assessment and has been used to develop a new modality for clinical alignment analysis.

Various parameters are related to sagittal balance and alignment change, including knee flexion through compensatory mechanisms, lower lumbar, and pelvic alignment. [7,8,9] The lower lumbar and pelvis are the main areas that determine the body’s sagittal balance in the spine. [10] Another important factor for sagittal balance is flexibility; lumbar flexibility (LF) is usually evaluated using a flexion-extension view, and pelvic flexibility (PF) is determined from the intrinsic pelvic morphology as pelvic incidence (PI). [11,12] Therefore, the flexibility of the lower lumbar and pelvis may be important factors in achieving a good outcome after TKA.

Therefore, the aims of this study were (1) to evaluate the relationship between lumbosacral flexibility and the effects of TKA on whole-body alignment, including spinal and lower limb alignment, and (2) to determine the prerequisites of the adjacent joints for successful TKA. The hypotheses of this study were that (1) patients with greater lumbosacral flexibility would have greater improvement in sagittal alignment after TKA, including lower limb and spinal alignment itself, and (2) reduced lumbosacral flexibility would cause the recurrence of lower limb deformity for the standing body balance.

## 2. Material and Methods

### 2.1. Demographics

Between February 2018 and September 2018, 116 patients (156 cases) who were available for serial (preoperative and 3-month and 1-year postoperative) whole-body EOS X-ray (EOS imaging, SA, Paris, France), imaging data, and flexion-extension lumbar radiograph were enrolled in this study. The exclusion criteria were as follows: (1) Secondary knee osteoarthritis with fracture malunion or non-union (1 case); (2) spinal fusion before or after TKA (12 cases); and (3) hip surgery before or after TKA (2 cases). Consequently, 101 patients (141 cases) were included in the final assessment.

Lateral whole-body EOS radiographs were used for the sagittal alignment evaluation. The neutral standing position was taken with the knee maximally extended and their tallest posture of hip joints and all the spine. The fist on the clavicle or face position was where the patient placed a hand on either side of the clavicle or face. INFINITT ver. 5.0.9.2 (INFINITT, Seoul, Korea), which can automatically measure up to 2 decimal places, was used for the radiological measurements. The knee joint disparities between postoperative passive motion and weight-bearing posture were assessed using the differences between the range of motion using a goniometer in the clinic and real measurements on lateral whole-body EOS radiographs. This study was approved by our Institutional Review Board in 2019 (B-1909/562-105).

### 2.2. Changes in Sagittal Alignment after TKA Considering Lumbosacral Flexibility

For the sagittal alignment evaluation, the sagittal hip–knee–ankle (HKA) angle, pelvic tilt (PT), sacral slope (SS), lumbar lordosis (LL), thoracic kyphosis (TK), and C7 plumb line-sacrum distance (SVA) were evaluated on the standing lateral whole-body EOS radiographs. For the lumbosacral flexibility, LF was evaluated on the flexion-extension lumbar radiographs and PF was evaluated on the lateral EOS radiographs.

**Figure 1:**
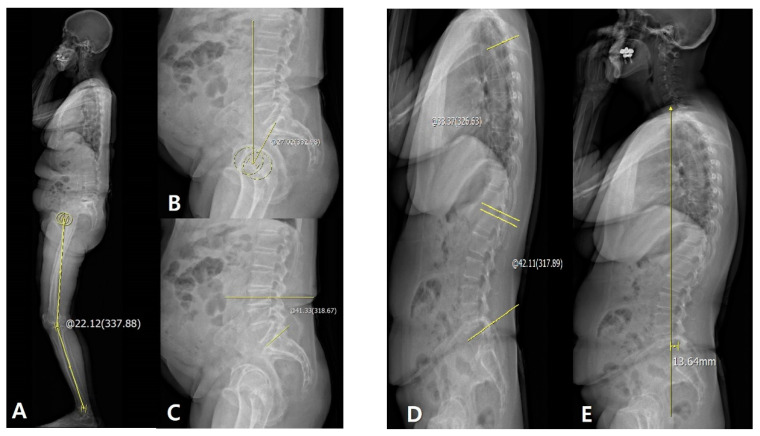
(**A**) Measurement of hip–knee–ankle (HKA) angle: +22.12°. (**B**) Measurement of PT: +27.02°. (**C**) Measurement of SS: +41.33°. (**D**) Measurement of TK and LL: +33.37° and +42.11°, respectively. (**E**) Measurement of C7 plumb line-sacrum distance: +13.64 mm. The sagittal HKA angle was defined as the angle between two lines: One joining the center of the femoral head and the center of the knee and the other joining the center of the knee and the center of the superiaor articular surface of the talus.

The sagittal HKA angle was defined as the angle between two lines: One joining the center of the femoral head and the center of the knee and the other joining the center of the knee and the center of the superior articular surface of the talus. The sagittal (HKA_Rt_–HKA_Lt)_ HKA_Rt–Lt_ angle was defined as the angle connecting the midpoints of each of the centers to the right and left HKAs (Figure 1A). PT was measured as the angle between the line joining the hip axis and the center of the S1 endplate and the reference vertical line (Figure 1B). SS was measured as the angle between the line along the S1 endplate and the reference horizontal line (Figure 1C). LL and TK were measured as the segmental angles of the spinal segments in lordosis (L1–L5) and kyphosis (T4–T12), respectively (Figure 1D). The SVA was measured as the distance between the vertical line from the midpoint of the C7 vertebral body and the posterior corner of S1. If the C7 plumb line was drawn in front of the sacrum, it was recorded as positive (+) (Figure 1E).

**Figure 2:**
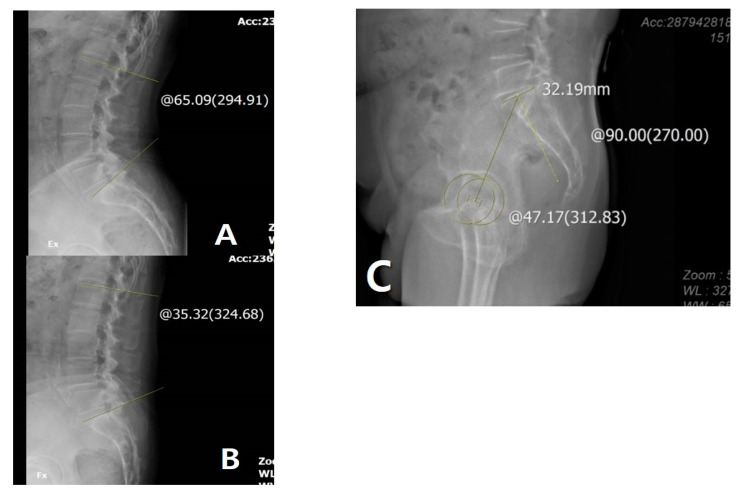
(**A**) Measurement of lumbar lordosis (LL) on extension view: 65.09°. (**B**) Measurement of LL on flexion view: 35.32°. (**C**) Measurement of pelvic incidence: 47.17°. The yellow line and circle are the measurement about lumbar lordosis and pelvic incidence

LF was measured as the absolute value of the difference between the angles of extension LL (Figure 2A) and flexion LL (Figure 2B). PF was defined as PI that was measured as the angle between the line joining the hip axis and the center of the S1 endplate and the line perpendicular to the S1 endplate (Figure 2C). The difference between the preoperative values and those at postoperative 3 months and 1 year was included in the serial assessments. Delta (Δ) was defined as the difference between the preoperative and postoperative values, and correlations between the lumbosacral flexibility and Δsagittal alignment parameters were evaluated.

### 2.3. Disparity after TKA Considering Lumbosacral Flexibility

The knee joint disparity between postoperative passive motion and weight-bearing posture was evaluated at postoperative 1-year. First, the angle of a passively full extended knee was measured manually in the clinic. Second, the sagittal HKA_Rt–Lt_ angle was measured on the lateral standing whole-body EOS radiographs. Then the difference between those two values was calculated. The correlation between disparity and lumbosacral flexibility was evaluated.

### 2.4. Statistical Analysis

All statistical analyses were performed with SPSS version 18.0 (IBM Corp., Armonk, NY, USA). Data are presented as mean and standard deviation. Pearson’s correlation analysis and linear regression analysis were performed to evaluate the presence of correlations between sagittal alignment change and lumbosacral flexibility, and the degree of disparity between clinically measured knee extension and standing knee alignment after TKA considering lumbosacral flexibility. Intra-class correlation coefficients were used to evaluate the intra- and inter-observer reliabilities. Results were considered statistically significant for *p*-value < 0.05.

## 3. Results

The mean age of the patients at the time of surgery was 70.55 years (range: 56–88 years). The inter- and intra-observer reliabilities for measuring the radiologic parameters were satisfactory, with mean kappa values of 0.90 and 0.83, respectively. The data of all parameters in the sagittal plane is summarized in Table 1.

### 3.1. Changes in Sagittal Alignment after TKA Considering Lumbosacral Flexibility

**Figure 3:**
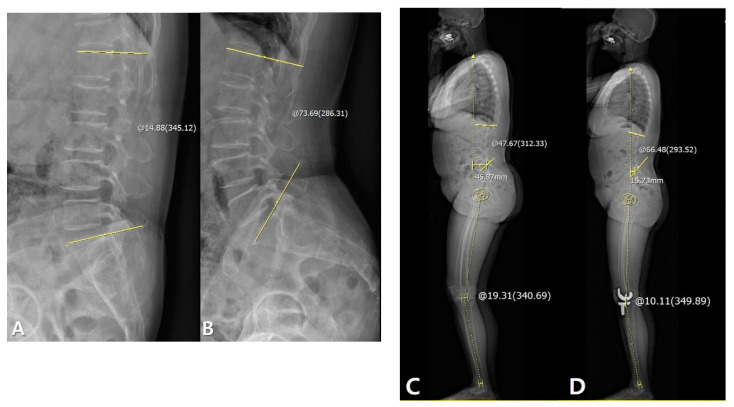
Patient with larger lumbar flexibility (LF) improved sagittal spinal alignment after TKA: Measurement of LF on flexion-extension view: 58.81°. (**A**) Measurement of LL on flexion view: 14.88°. (**B**) Measurement of LL on extension view: 73.69°, improved spinal alignment increased LL and decreased line-sacrum distance (SVA). (**C**) Measurement of the preoperative LL: 47.67° and SVA: 45.87 mm. (**D**) Measurement of postoperative LL: 66.48° and SVA: 15.73 mm. The yellow line and circle are the measurement about lumbar lordosis, SVA and sagittal HKA.

The correlations between the LF and other sagittal parameters are summarized in Table 2. LF was significantly correlated with ΔLL and ΔSVA between preoperative and 1-year postoperative data (LL: *p* = 0.039, SVA: *p* = 0.040; Pearson correlation coefficient (PCC): −0.206 and 0.205, respectively). However, TK, PT, SS, and HKA were not correlated with the LF. Linear regression analysis for the identification of a cause–result relationship revealed that larger LF significantly increased LL and decreased SVA after TKA (*p* = 0.039, β = −0.155 and *p* = 0.040, β = 0.681, respectively) (Table 2, Table 3 and Figure 3).

**Figure 4:**
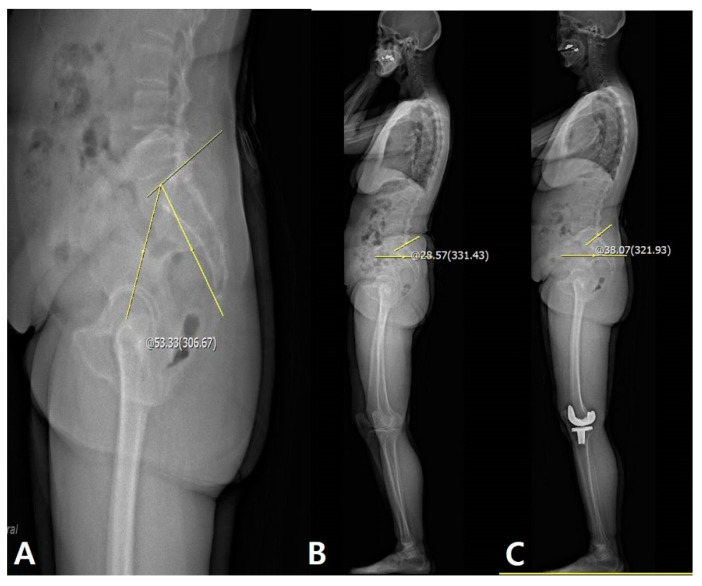
Patient with larger pelvic flexibility (PF) improved sagittal pelvic alignment, such as increased SS, after TKA. (**A**) Measurement of PF: 53.33°. (**B**) Measurement of preoperative SS: 28.57°. (**C**) Measurement of postoperative SS: 38.07°. The yellow line and circle are the measurement about pelvic incidence and sacral slope.

The correlations between the PF and other sagittal parameters are summarized in Table 3. Although there were correlations between PF and ΔSS between preoperative and 1-year postoperative data (*p* < 0.001, PCC: −0.362), other sagittal parameters were not correlated with the PF. Linear regression analysis for the identification of cause–result relationships revealed that larger PF significantly increased SS after TKA (*p* < 0.001, β = −0.208) (Table 3 and Table 4, Figure 4).

### 3.2. Disparity after TKA Considering Lumbosacral Flexibility

**Figure 5:**
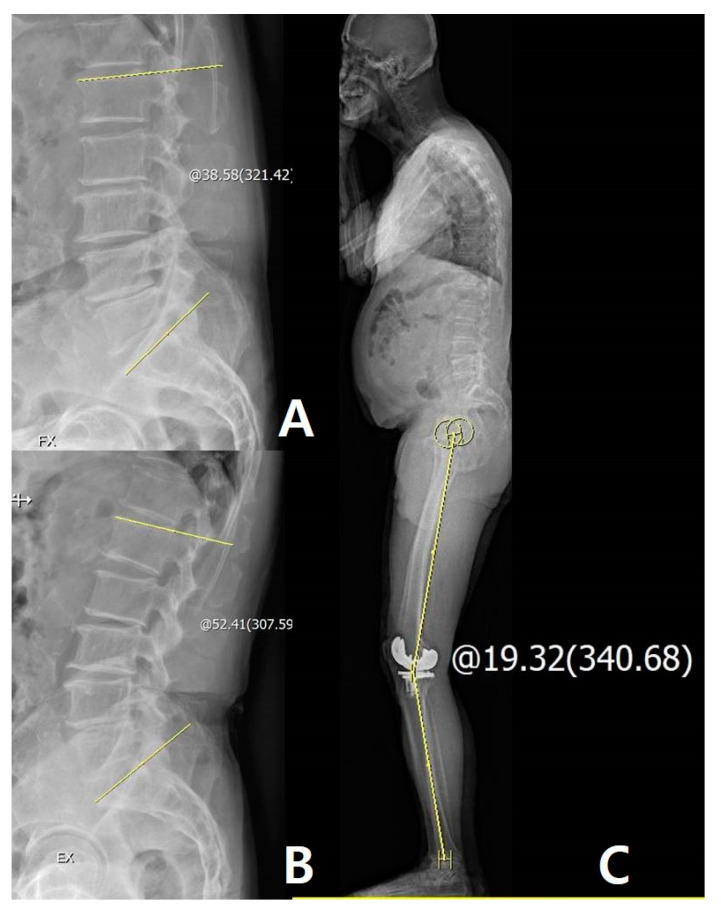
Patients with lesser LF have disparities in the knee joint between passive extension and real standing extension after TKA. (**A**) Measurement of LL on flexion view: 38.58°. (**B**) Measurement of LL on extension view: 52.41°. (**C**) Measurement of disparity is 19.32° with passive full extension. The yellow line and circle are the measurement about lumbar flexibility and sagittal HKA.

Correlations between the disparity of the clinical and radiological extension status and lumbosacral flexibility 1 year postoperatively are summarized in Table 5. Although there were correlations between the disparity and LF at postoperative 1 year (*p* = 0.005, PCC = −0.275), PF was not correlated with the disparity. Linear regression analysis demonstrated that LF was significantly associated with the disparity between clinical and radiological extension status (*p* = 0.005, β = −0.205) (Figure 5).

## 4. Discussion

The principal findings of this study were as follows: First, the patients with greater preoperative LF achieved better lower limb and spinal alignment, such as increased LL, that improved SVA after TKA. Furthermore, the patients with greater preoperative PF had better pelvic alignment, such as increased SS after TKA. Second, the LF was related to the disparity of the extension status between clinically measured and actual weight-bearing posture, and the disparity was predominantly observed in patients with lower LF. Therefore, our two hypotheses were accepted.

Several trials have attempted to identify the relationship between the axial and lower limb alignments. Murata et al. [3] described “knee-spine syndrome” and stated that there may be an associated loss of LL if a patient has a fixed flexion contracture of the knee. Lee et al. [13] reported that a change in flexion contracture after TKA had an effect on pelvic parameters, such as SS. However, few studies have attempted to verify the effect of spinal flexibility on the axial and limb alignment. Therefore, our study has clinical relevance in that it demonstrates that spinal flexibility is an important factor for improved spinal and lower limb alignment after TKA. In other words, these findings imply that the flexibility of each site can give a good effect of each site after TKA.

**Figure 6:**
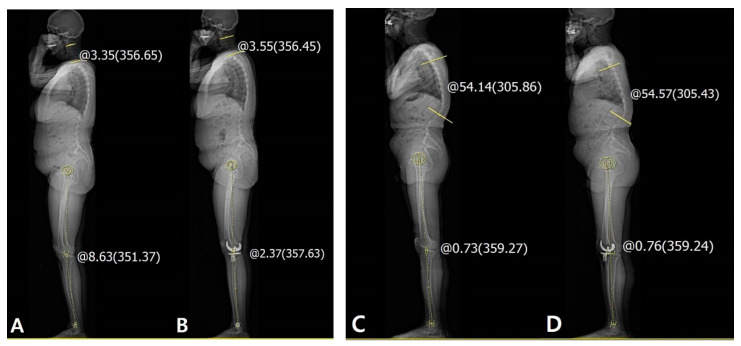
Cervical deformity showed little effect on the lower limb and spinal alignment itself. (**A**) Preoperative cervical lordosis: 3.35° and HKA angle: 8.63°. (**B**) Postoperative cervical lordosis: 3.55° and HKA angle: 2.37°. Thoracic deformity showed little effect on the lower limb and spinal alignment itself. (**C**) Preoperative thoracic kyphosis: 54.14° and HKA angle: 0.73°. (**D**) Postoperative thoracic kyphosis: 54.57° and HKA angle: 0.76°. The yellow line and circle are the measurement about sagittal HKA and cervical lordosis and thoracic kyphosis.

Interestingly, some patients demonstrated a tendency to stand with knee flexion even if the fixed knee flexion contracture was corrected and there was no flexion contracture in the clinical measurement. From our study, we believe that this disparity was mostly related to the reduced LF. Cervical or thoracic deformity showed limited effect on the lower limb or spinal alignment itself (Figure 6). Therefore, preoperative evaluation of the LF would be important for successful TKA. Since this disparity is associated with lumbar flat back or stooping during standing as a compensation mechanism, [14,15,16] it can be problematic in that the flexible knee extension status can become a fixed flexion contracture with time.

Knee surgeons should operate by considering the alignment of the whole-body since all joints are inter-related and can compensate each other. This concern was first raised in spinal studies, and EOS has subsequently enabled assessment of the whole-body alignment. [17] Most patients with knee problems complain about spinal problems seriously, but rarely complain of hip or ankle problems. In addition, hip and ankle problems show little deformity or symptom that requires surgical intervention, especially in Asian patients. This may be related to the stability of the hip and ankle joint compared to the unstable knee joint. [3] EOS system involves two perpendicular X-ray beams moving vertically, with the patient standing in the center of the scanning booth and the entire body, or part of it, is scanned with simultaneous projections in two perpendicular planes without magnification. [17,18] Accordingly, the EOS system is able to evaluate the effect on adjacent joints without magnification after TKA.

Spinal flexibility decreases with age and the spine degenerates. The main areas of sagittal balance in the body are the lumbar and pelvis, and this lumbosacral flexibility can be evaluated separately using LF and PF. [7,10] Most spine surgeons use forward bending and backward return for checking spinal mobility, [19] and changes in lumbar lordosis in the lumbar flexion-extension view reflect sagittal LF. [11,12] PF, determined as PI that is intrinsic value in each person and the importance of the formula, SS + PT = PI is that the ability to change PT or SS for compensation of sagittal alignment is determined by the size of the PI. [7,8] Therefore, we evaluated using the difference of the LL in the lumbar flexion-extension view and PI on the EOS lateral X-ray.

The clinical relevance of this study is that fixed spinal deformity, especially lumbar sagittal alignment, should be corrected before TKA or we should give information to patients on the development of knee flexion contracture if patients have asymptomatic fixed spinal deformity. There are some limitations to be considered. First, the clinical and radiological measurements of the extension status were performed using different tools. Therefore, there was a tendency that the radiological value was comparatively larger than that of the clinical measurement. We believe that the radiologic measurement used in this study with the EOS system is more appropriate for accurate quantitative analysis than the goniometric measurement in the clinic. Second, the follow-up period was short and we were unable to verify whether this disparity represents a true flexion contracture over time. Third, we only performed objective measurements of parameters, and changes in the apparent clinical symptoms, such as walking discomfort and lower back pain, were not included in this study. Fourth, the current study focused only on sagittal alignment; however, there was no specific correlation between coronal spinal and knee alignment in our previous study. Therefore, we did not perform an evaluation of the coronal parameters. In addition, knee joint parameters such as posterior tibial slope were not considered. Generally, we performed posterior stabilizing TKA within 5 degrees posterior slope. Therefore, this effect can be neglected because most patients had similar slopes.

## 5. Conclusions

LF was an important factor for improved spinal and lower limb alignment after TKA. Additionally, a lower LF may develop disparity in the knee joint between passive extension and standing extension status. Therefore, surgeons should consider spinopelvic alignment, including lower limb alignment, preoperatively for predicting possible changes in the whole-body alignment following TKA.

## Figures and Tables

**Table 1:** Data of all sagittal parameters.

Parameter	Preoperative	Postop 3 Months	Postop 1 Year	*p*-Value
Clinical parameters (*n* = 101)				
Flexion contracture (°) Rt	9.85 ± 6.26		0.24 ± 1.09	<0.001
Flexion contracture (°) Lt	10.19 ± 5.65		0.29 ± 1.19	<0.001
Radiologic parameters (*n* = 101)				
Sagittal HKA angle (°)	10.57 ± 5.81	7.22 ± 5.71	5.52 ± 6.22	<0.001
Thoracic kyphosis (°)	33.82 ± 10.43	33.07 ± 10.66	31.29 ± 11.03	<0.001
Lumbar lordosis (°)	46.42 ± 12.47	48.21 ± 11.47	48.62 ± 12.45	0.004
SVA (mm)	25.80 ± 33.54	23.45 ± 36.99	23.01 ± 33.79	0.018
Pelvic tilting (°)	21.36 ± 8.73	18.87 ± 8.05	18.68 ± 8.07	<0.001
Sacral slope (°)	34.70 ± 7.96	37.61 ± 8.39	35.73 ± 8.00	0.071

HKA: Hip–knee–ankle, Postop: Postoperative, Rt: right, Lt: Left, SVA: C7 plumb line-sacrum distance. The values are presented as mean and standard deviation. The statistical significance was set at *p* < 0.05. The *p*-value was derived from comparison between the preoperative and 1-year postoperative values.

**Table 2:** Correlation analysis between lumbar flexibility and sagittal parameters.

Parameter	Preoperative— Postop 3 Months	Preoperative— Postop 1 Year
ΔThoracic kyphosis (°)		
PCC	0.161	0.092
Significant probability	0.108	0.361
ΔLumbar lordosis (°)		
PCC	−0.165	−0.206
Significant probability	0.099	0.039
ΔC7 plumb line-sacrum distance (mm)		
PCC	0.055	0.205
Significant probability	0.582	0.040
ΔPelvic tilting (°)		
PCC	0.148	0.013
Significant probability	0.138	0.897
ΔSacral slope (°)		
PCC	−0.034	−0.032
Significant probability	0.732	0.749
ΔHKA angle (°)		
PCC	0.023	0.113
Significant probability	0.820	0.261

HKA: Hip–knee–ankle, Postop: Postoperative, PCC: Pearson correlation coefficient, Δ: Preoperative value–postoperative value.

**Table 3:** Subgroup analysis to identify the cause–result relationship using linear regression analysis.

Independent Variable	Dependent Variable	Preoperative— Postop 1 Year	
		Regression Coefficient (*β*)	*p*-Value
LF	ΔLL	−0.155	0.039 *
LF	ΔSVA	0.681	0.040 *
PF	ΔSS	−0.208	<0.001*

The statistical significance was set at *p* < 0.05. * *p*-value < 0.05. Postop: Postoperative, Δ: Preoperative value—postoperative value, LF: Lumbar flexibility; PF: Pelvic flexibility, LL: Lumbar lordosis, SVA: C7 plumb line-sacrum distance, SS: Sacral slope.

**Table 4:** Correlation analysis between pelvic flexibility and sagittal parameters.

Parameter	Preoperative— Postop 3 Months	Preoperative— Postop 1 Year
ΔThoracic kyphosis (°)		
PCC	−0.127	0.049
Significant probability	0.206	0.623
ΔLumbar lordosis (°)		
PCC	0.182	−0.071
Significant probability	0.069	0.478
ΔC7 plumb line-sacrum distance (mm)		
PCC	−0.024	−0.050
Significant probability	0.809	0.619
ΔPelvic tilting (°)		
PCC	0.024	0.057
Significant probability	0.813	0.575
ΔSacral slope (°)		
PCC	−0.102	−0.362
Significant probability	0.309	<0.001
ΔHKA angle (°)		
PCC	0.068	0.134
Significant probability	0.501	0.180

HKA: Hip–knee–ankle, Postop: Postoperative, PCC: Pearson correlation coefficient, Δ: Preoperative value–postoperative value.

**Table 5:** Correlation analysis between disparity and lumbosacral flexibility.

Lumbosacral Flexibility	Correlation
Lumbar flexibility (°)	
PCC	−0.275
Significant probability	0.005
Pelvic flexibility (°)	
PCC	0.075
Significant probability	0.456

PCC: Pearson correlation coefficient.
